# Geospatial analysis of healthcare and older adult care institutions in Wuhan: a multimethod approach to assessing spatial equity

**DOI:** 10.3389/fpubh.2025.1580630

**Published:** 2025-08-05

**Authors:** Bo Li, JingYi Long, Yi Li

**Affiliations:** ^1^School of Public Administration, China University of Geosciences, Wuhan, China; ^2^Wuhan Mental Health Center, Wuhan, China; ^3^Affiliated Wuhan Mental Health Center, Tongji Medical College of Huazhong University of Science and Technology, Wuhan, China

**Keywords:** spatial equity, healthcare-eldercare integration, GIS analysis, urban–rural disparities, service accessibility

## Abstract

**Background:**

Achieving spatial equity in healthcare and older adult care services is critical for ensuring fair and effective service access among aging populations. In rapidly urbanizing cities like Wuhan, the spatial distribution of facilities directly influences accessibility and integration outcomes. While existing research has primarily focused on service demand, the spatial distribution of medical and older adult care institutions which is a key factor for achieving effective integration remains underexplored.

**Method:**

The current study classifies medical and older adult care institutions into four categories and employs multiple spatial analysis methods such as Ripley’s K-function (K), the geographical concentration index (G), the imbalance index (S), and kernel density analysis using Geographic Information System (GIS) to examine their spatial distribution in Wuhan. The spatial characteristics, distribution patterns, and interrelationships among these institutions are examined in the context of Wuhan, China. These spatial analysis methods are employed to assess disparities in the geographic distribution of institutions, highlighting spatial inequity between urban and peripheral areas.

**Results:**

(1) Ripley’s K-analysis reveals significant spatial clustering across all four institution categories, with observed *K*-values exceeding expected thresholds and high confidence levels.

(2) The geographical concentration index $G_{0}\;$= 27.73, with *G* values surpassing this threshold for all four categories, indicates a pronounced spatial concentration.

(3) The imbalance index (S > 0) indicates considerable disparities in the spatial distribution of resources across all categories.

(4) Kernel density analysis identifies a strong concentration of institutions in central urban areas, highlighting notable urban–rural disparities in service accessibility.

**Conclusion:**

The results reveal significant spatial clustering and disparities in the distribution of older adult care institutions, highlighting challenges in equitable resource allocation and urban planning. Ultimately limiting the accessibility, availability and equity of services for the older adult population. To address these disparities, policymakers should prioritize spatial equity in planning decisions, ensuring balanced service distribution that supports healthy aging objectives and the goals of “Healthy China 2030. Such effort are essential to improving system efficiency and enhancing the quality of life for China’s aging population, both in Wuhan and across the nation.

## 1 Introduction

Spatial equity in the distribution of healthcare and older adult care services is a growing concern in aging societies, especially in urbanizing regions like Wuhan. While demographic aging increases the demand for integrated care, the spatial imbalance in facility locations can create major barriers to accessibility and quality of service for older adults (1, 2). These shifts have led to growing demand for public facilities and services, particularly among older adults, who are heavily dependent on healthcare services (3, 4). Providing professional, planned, and integrated healthcare for older adult is essential for addressing their growing needs and ensuring their quality of life (5). Unfortunately, the supply of healthcare services often lags behind this rising demand, resulting in persistent gaps in healthcare infrastructure, particularly in developing countries (6).

Promoting healthy aging has become a global priority and a key research area in aging societies. It is considered a vital strategy for addressing the multifaceted challenges of population aging (7, 8). According to the results of China’s seventh census, by the end of 2020, individuals aged 60 and older constituted 18.7% of the population (264 million), while those 65 and older accounted for 13.5% of the population (191 million) (9). This aging trend is expected to deepen, posing significant long-term challenges to socioeconomic development and population dynamics (10). The growing burden of chronic illnesses like diabetes among older adults has led to a heightened need for sustained healthcare services, emphasizing the importance of integrated healthcare planning in china (11). In response, China has introduced policies aimed at improving equity, accessibility, and sustainability of healthcare services for its aging population. Although China has made some progress in this regard over the past decade (12), structural challenges remain in planning and delivering older adult services in urban areas, leaving the supply–demand gap unaddressed (13). Despite the focus on healthcare-eldercare integration in recent policy and academic discourse, limited research has addressed whether these services are equitably distributed across space. Spatial equity-grounded in the principles of spatial justice and equity of access-provides a critical framework to examine how geographic disparities impact the effectiveness of service integration. This study seeks to bridge that gap.

China’s older adult care industry, particularly in first-tier cities, has been developed significantly since 2012. Yet, even in these developed regions, existing senior care facilities often fail to meet planning requirements due to mismatches between supply and demand (14). The fragmented services, unplanned allocation, socioeconomic status, and varying levels of technical support exacerbate the problem (15, 16). Moreover, the insufficient integration of medical and nursing resources contributes to spatial imbalances, depriving older individuals in certain areas of adequate care and hindering the integration of healthcare and eldercare systems (17, 18). Addressing these challenges requires a comprehensive strategies that incorporate equitable planning and integrated services and fully meet the needs of older adult, comprehensive solutions that promote integrated healthcare and older adult care are needed (19). Spatial equity refers to the fair and just distribution of resources and services across geographic space, ensuring that individuals have comparable access regardless of where they reside. Rooted in the theory of spatial justice, it emphasizes that public services-including healthcare and older adult care-should not be disproportionately concentrated in certain regions while excluding others. In the context of an aging society, spatial equity is crucial to mitigate geographic disparities in access to critical services.

In the field of urban planning, recent studies emphasize spatial equity in public service distribution, particularly the concept of spatial justice has been applied to analyze the fairness of healthcare facility locations (20, 21). For instance, studies using location-allocation modals have demonstrated how strategically adding new healthcare facilities can enhance spatial accessibility, supporting more effective and equitable health planning in urban areas of china (22). These approaches provide valuable insights for effectively integrating healthcare and older adult care planning within the broader context of urban development. This integration benefits the aging population, alleviate healthcare burdens, and optimized medical institutional capacity (23, 24). Healthcare-older adult care integration refers to the coordinated delivery of medical and long-term care services tailored to the needs of older adults. It typically involves two main models: where care services are co-located within medical institutions, and the contractual model, where services are linked via partnerships or agreements. While integration seeks to improve continuity of care, its effectiveness is influenced by how equitably resources are distributed spatially. Therefore, analyzing integration from a spatial equity lens is essential to understand who benefits and who may be left underserved. Continuously improving and expanding support services for older adult is crucial to ensuring a dignified and healthy experience (25).

Accessibility, a key concept in this study, has been examined across various dimensions, including the quantity, proximity, and quality of services as described by some researchers in the literature (26). Studies in healthcare accessibility highlight the critical role of geographic distribution emphasizing the importance of proximity and timely access, especially for emergency medical care where timely intervention is crucial (27–29). Notably, spatial accessibility is influenced by multiple factors beyond just the location of healthcare facilities, including how populations are distributed, the availability of medical resources, and the structure of transportation network (30). Therefore, it is important to ensure the rational configuration of healthcare facilities and promote high accessibility to meet the older adults effectively (31).

Recent studies using Geographic Information System (GIS) techniques have mapped healthcare accessible and revealed significant disparities in service availability across urban and rural areas (32, 33). Additionally, research has examined the spatial patterns of senior care institutions and their correlation with demographic changes (34). However, there is a notable gap in the integrated spatial analysis of both healthcare and senior care institutions particularly, in the context of rapidly aging cities in developing countries. While the concept of healthcare-eldercare integration has gained attention (35), many existing studies have focused primarily on organizational aspects, with limited focus on the spatial dimensions such as resource distribution and accessibility. This gap becomes even more critical as policies promoting aging-in-place emphasize the end for coordinated healthcare and older adult care services (36).

Most prior studies employ single-method approaches such as the geographic concentration index (37–39), or incorporated kernel density analysis (40–42), which provides valuable insights, but often fail to capture the complex interplay between different facility types. More comprehensive spatial analysis approaches, such as the Index of Spatial Accessibility (ISA), have been employed to healthcare accessibility studies (43) but remain underutilized in healthcare-eldercare integration research.

This study aims to address these gaps by investigating the spatial equilibrium of medical institutions and senior care institutions in Wuhan, China, using a multimethod approach. We hypothesize significant spatial inequality in the distribution of these facilities, affecting accessibility and quality of care for older adult individuals. The objectives of current research are as follows:

1. Analyze the spatial equity of healthcare and older adult care institutions in Wuhan using GIS-based spatial methods.
2. Evaluate disparities in service accessibility between urban and rural areas.
3. Provide evidence-based recommendations to inform equitable and integrated planning.

The innovation of this study lies in its integrated approach to analyzing both healthcare and older adult care institutions employing multiple spatial analysis methods, including combining multi-distance spatial cluster analysis (Ripley’s K function), the geographic concentration index (G), the imbalance index (S), and kernel density analysis. This enables a more comprehensive understanding of spatial distribution patterns and their implications for healthcare-eldercare integration.

Ultimately, the findings contributes to theoretical and practical knowledge on spatial equity, offering actionable insights for urban planners and policymakers to optimize service distribution and support aging-in-place strategies.

## 2 Research site and database

### 2.1 Research site

Wuhan, the provincial capital of Hubei Province, hold significant strategic and administrative importance in China. It is designated as a national central city and serves as the core hub of the Yangtze River Belt. Furthermore, it fulfills multiple roles, including serving as a national business center, a national science and technology innovation center, a national trade and logistics hub, and an international communication and financial center. Its designation as a strategic pivot city for the revitalization of central China underscores its vital role in regional and national development.

**Figure 1:**
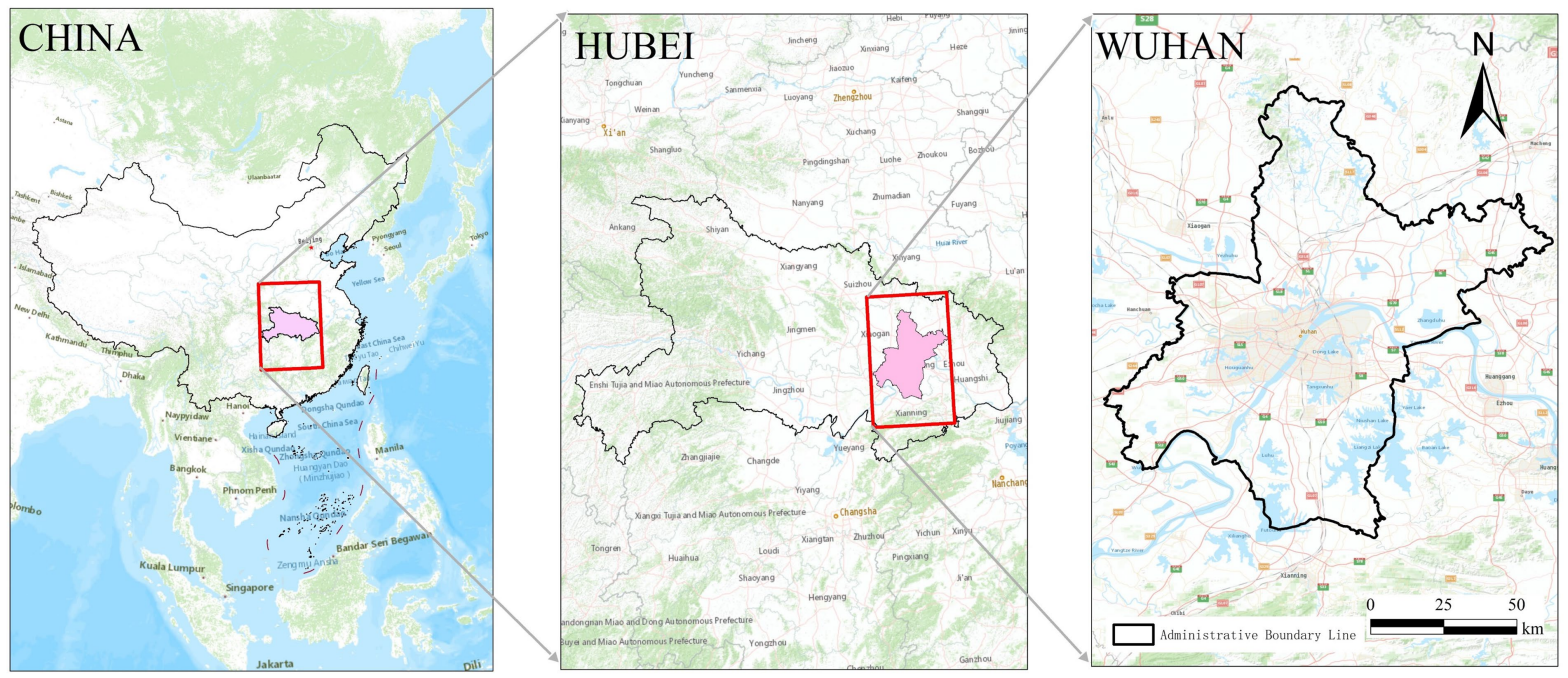
Research site.

According to China’s seventh national population census, the resident population of Wuhan is 12.33 million, of whom 17.23% are aged 60 or older, Wuhan presents a representative case for urban aging in China. Administratively, the city comprises 13 districts and covers 8,569.15 km^2^, encompassing diverse urban and suburban environments (Figure 1).

### 2.2 Concept definition

This paper primarily utilizes Geographic Information System (GIS) techniques to conduct a spatial analysis of the distribution of medical and senior care institutions in Wuhan. The research focuses on two categories:

- Medical institutions: Classified into tertiary hospitals, secondary hospitals, and community health service centers based on national standards.
- Senior care institutions: Including nursing homes, welfare hoes, and older adult apartments with at least 10 beds.

These classifications allow for meaningful comparison of spatial distribution and service accessibility across healthcare and eldercare systems (Table 1), as detailed in Supplementary Table S1.

**Table 1:** Categories and properties of the research objects.

Category	Subcategory	Service focus	Target users	Operational nature
Medical institutions	Tertiary Hospital Level II Hospital Community Health Service Center	Social medical services	Society at large scale	Government-run Social start-up
Senior care institutions	Nursing Homes Welfare Homes Older adult Apartments	Social public services	Seniors	Government-run Social start-up

### 2.3 Data resources

This study focuses on data pertaining to medical institutions, senior care institutions, and associated points of interest (POI) within Wuhan.

#### 2.3.1 Senior care institutions

The data on senior care institutions were obtained from the Senior Care Service Department of the Wuhan Civil Affairs Bureau. According to these data, Wuhan hosts 245 senior care institutions categorized as follows:

- 14 government-operated senior care institutions in urban areas.
- 52 rural senior care institutions serving less densely populated regions.
- 179 social start-up senior care institutions providing privately run services.

#### 2.3.2 Medical institutions

Information regarding medical institutions was extracted from the official website of the Wuhan Health Administration Committee. The dataset included a list of secondary and tertiary medical institutions as well as primary medical facilities such as community health service centers, specifically:

- 65 tertiary medical institutions delivering advanced medical care and expertise
- 68 secondary medical institutions offering intermediate healthcare services.
- 205 community health service centers in Wuhan focusing on primary healthcare at the local level.

To determine the quality of medical institutions, parameters such as the technical level, service capability, and adherence to quality and safety standards were considered. High-quality medical institutions were defined as Secondary and tertiary medical institutions. Moreover, specialized medical institutions such as children’s hospitals, maternity hospitals, anorectal hospitals, and plastic surgery hospitals were excluded from the analysis to maintain the uniformity in the dataset. Consequently, the refined dataset comprised:

- 31 tertiary medical institutions
- 42 secondary medical institutions
- 205 community health service centers
- 245 senior care institutions

Table 2 summarizes the distribution of medical institutions and senior care facilities across Wuhan’s districts, providing a comprehensive overview of the resource landscape detailed in Supplementary Table S2.

**Table 2:** Distribution of medical and older adult care institutions in each district of Wuhan.

Administrative district	Tertiary care	Secondary MI	Community HC	MI above SI	Total MI	Older adult CI
Jiang’an	5	3	17	8	25	27
Wuchang	5	7	18	12	30	23
East–West Lake	3	2	12	5	17	10
Jiangxia	3	1	26	4	30	16
Qiaokou	3	3	11	6	17	24
Hanyang	2	2	10	4	14	24
Huangpi	2	2	20	4	24	35
Jianghan	2	7	13	9	22	15
Qingshan	2	2	14	4	18	9
Hongshan	1	8	24	10	33	17
Hannan	1	2	10	3	13	6
Xinzhou	1	1	18	2	20	26
Caidian	1	2	12	3	15	13
Total	31	42	205	73	278	245

MI, medical institute; HC, health care; SI, secondary institute; CI, community institute.

In addition to the spatial count of institutions, we collected district-level population data-particularly the older adult population (aged 60 and above)-from the Wuhan Bureau of Statistics (latest available year: 2020/2021). To normalize service availability, we calculated the number of institutions per 10,000 older adult residents for each district. This adjustment helps assess resource equity relative to need rather than absolute distribution alone.

### 2.4 Data exclusion criteria and handling of missing data

To ensure data integrity and consistency, institutions were excluded if their addresses could not be geocoded accurately using Baidu Maps or if their operational status could not be confirmed via official sources (e.g., Civil Affairs Bureau and Health Administration websites). Specifically, facilities were removed from the dataset if they:

- Lacked complete or verifiable address information,
- Had ambiguous classification (e.g., hybrid institutions not fitting defined categories), or
- Were inactive or listed as “temporarily closed” in government records.

The impact on overall sample representativeness was minimal, as the excluded facilities were geographically scattered and did not disproportionately affect any specific district. Sensitivity checks confirmed that key spatial patterns remained consistent when including or excluding borderline cases.

## 3 Method

This study uses a multidimensional approach, including multi-distance spatial cluster analysis using Ripley’s K function, the geographical concentration index (G), the imbalance index (S), and kernel density estimation to analyze the spatial distribution balance across different categories of institutions.

**Figure 2:**
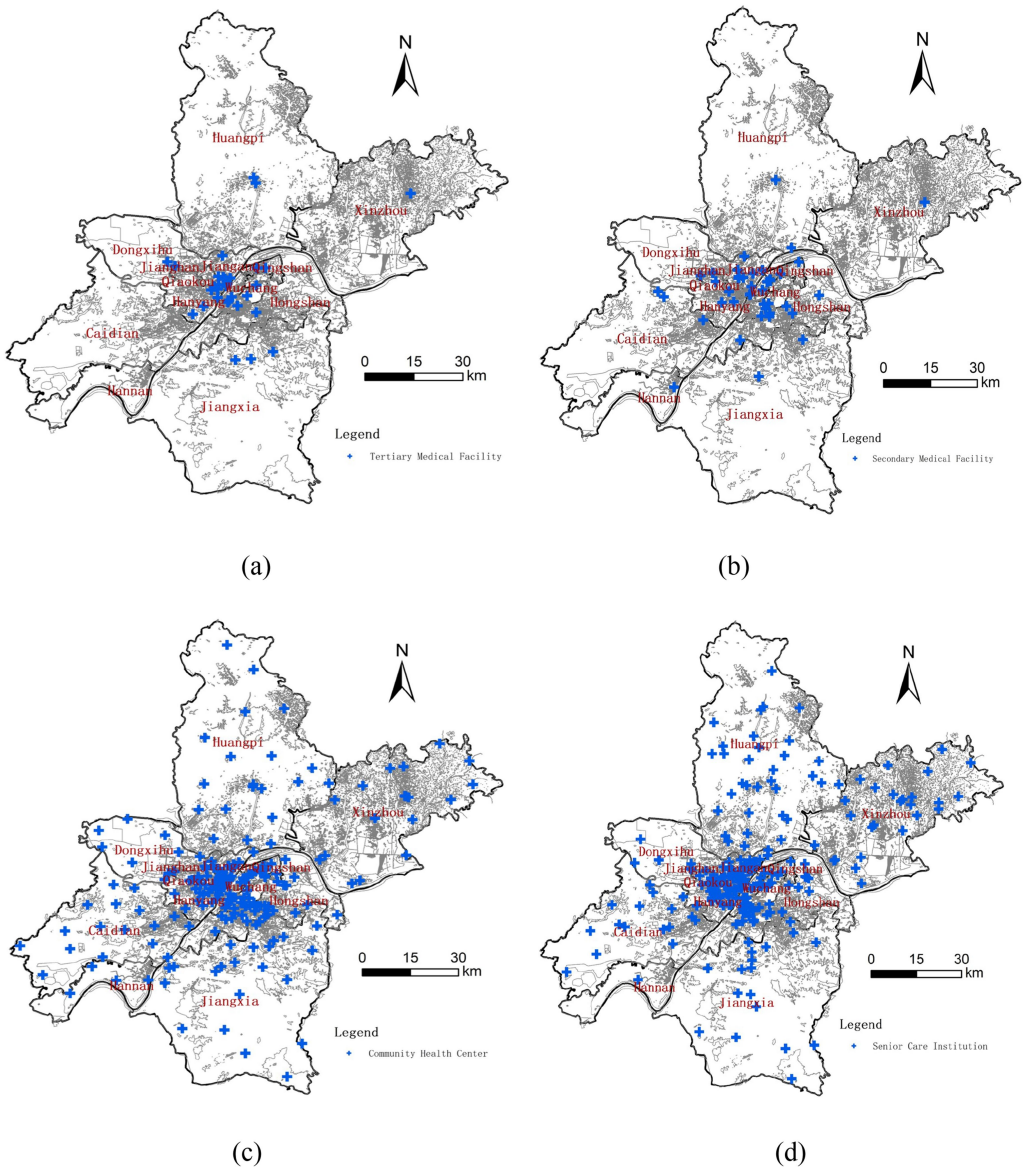
Spatial distribution of institutions by category. **(a)** POIs of tertiary medical facility. **(b)** POIs of secondary medical facility. **(c)** POIs of community health center. **(d)** POIs of senior care institution.

A total of 523 institutions, consisting of senior care and medical facilities were analyzed. The points of interest (POIs) for these institutions were obtained via the “coordinate picker” function of the Baidu Maps, with cross-verifying of facility names and addresses to ensure data accuracy. The collected data was then categorized into four primary types: (a) tertiary medical facilities, (b) secondary medical facilities, (c) community health services, and (d) senior care institutions (Figure 2).

### 3.1 GIS software and potential biases

All spatial analysis were conducted using ArcGIS 10.8. The spatial coordinate system used was WGS 1984, with projections converted to UTM Zone 49 N for spatial analysis. Data layers were cleaned, digitized, and managed within the ArcMap environment.

### 3.2 Data validation and potential biases

To ensure accuracy:

- POIs were double-checked against multiple official sources.
- Facilities with incomplete or unverifiable data were excluded.
- Spatial outliers (e.g., institutions located far outside district bounds) were investigated and corrected where needed

Potential biases may include:

- Temporal mismatch in data collection from different sources.
- Possible underreporting or classification inconsistency, particularly in private or rural facilities.
- Reliance on Baidu Map’s geocoding, which may introduce slight spatial inaccuracies.

### 3.3 Spatial analysis methods

#### 3.3.1 Ripley’s K function

Ripley’s K function (Equation 1) is a widely used method for spatial point pattern analysis that can be used to detect spatial clustering or dispersion of objects across varying spatial scales various scales (44). In recent years, it has been extensively applied in the analysis of complex spatial data (45). In this study, we employed Ripley’s K function, to examine the spatial distribution patterns of medical and older adult care institutions in Wuhan, and the K function is mathematically defined as:


(1) $$L(d)=\sqrt{\frac{A\sum_{i=1}^{n}\sum_{j=1j\neq 1}^{n}k_{i,j}}{\mathrm{\pi n}(n-1)}}\;$$

Where:

- d = distance threshold
- n = number of points
- A = study area
- $k_{\mathrm{ij}}$ = 1 if distance between *i* and j < *d*; else 0

A *K*-value above the expected indicates clustering; below indicates dispersion. Results are interpreted using confidence envelopes generated within ArcGIS.

When applying Ripley’s K function within GIS systems, specified confidence intervals produces additional output fields such as, LwConfEnv (low-value confidence interval) and HiConfEnv (high-value confidence interval), observations of *K* lead to the following results.

The observed value of *K*>. The expected value of *K* indicates that the distribution is clustered to a greater degree than a random distribution within a certain distance.

- The observed value of *K*< the expected value of *K*, which indicates that the distribution is more discrete than a random distribution within a certain distance.
- K observations> the HiConfEnv value, which indicates that the spatial clustering within a certain distance is statistically significant.
- K observations< the LwConfEnv value indicates that the spatial dispersion is statistically significant within a certain distance.

#### 3.3.2 Geographic concentration index (G)

The geographic concentration index (G) was first introduced by Florence as a metric to measure the spatial concentration of geographic elements (46). This index has been widely utilized in recent research to analyze the spatial agglomeration of economic activities (47, 48). In this study, the G index is used to evaluate the spatial distribution concentration of various types of institutions, and its formula is defined as:


(2) $$G=100\sqrt{\sum_{i=1}^{n}(\frac{x_{i}}{T})}2$$

Where:

- $X_{i}$ = number of institutions in district *i*
- T = total number of institutions
- n = number of districts

To determine whether the observed spatial distribution is more clustered than a hypothetical even distribution, the following formula for an evenly distributed scenario is used:


(3) $$G=G_{0}=100\sqrt{\sum_{i=1}^{n}(\frac{1}{n})2}$$

- $G>G_{0}$: implies spatial concentration.

#### 3.3.3 Imbalance index (S)

The imbalance index (S) (Equation 4) reflects the degree of spatial disparity in the distribution of institutions within a specific area. It was derived from the inequality measurement method proposed by Gini, this index has gain significant application in public health studies in recent years (49). In this study, the imbalance index measures the extent to which each type of institution is evenly or unevenly distributed spatially. The formula is as follows:

Where:


(4) $$S=\frac{\sum_{i=1}^{n}Y_{i}-50(n+1)}{100n-50(n+1)}$$

- n = total districts
- $Y_{i}$ = cumulative percentage of institutions sorted in descending order
- 50(n+1) = sum of the cumulative percentage if the institutions were perfectly evenly distributed across all districts.

In this case, each district would have $(\frac{100}{n})\%$ of the study subjects, and the cumulative percentage would form an arithmetic sequence. 100n represents the maximum possible sum of cumulative percentages, which would occur if all institutions were concentrated in a single district. S represents a cumulative percentage value ranging from 0 to 1. When S=0, the research objects are evenly distributed in each region, conversely, when S=1, the research objects are all concentrated in one area.

This imbalance index offers a precise metric to evaluate the spatial inequality, highlighting region with disproportionate concentrations of medical and senior care institution. Such information is crucial for optimizing resource allocation and addressing spatial imbalances in public health infrastructure.

#### 3.3.4 Kernel density analysis (KDE)

Kernel density analysis (Equation 5) is a non-parametric statistical method that is used to estimate the spatial density of point patterns. As an effective spatial density estimation technique, this method has gained wide applicability across diverse fields, including hotspot prediction (50), and can reflect the spatial density of the study object. In this study, the kernel density analysis is integrated with GIS technology to visualize the spatial sparseness, senior care institutions, spatial distribution of medical, effectively representing their density and sparseness across the region. The mathematical expression for kernel density analysis is shown as follows:

Where:


(5) $$f_{\left(x\right)}=\frac{1}{\mathrm{nh}}\sum_{i=1}^{n}k(\frac{x-x_{i}}{h})$$

- k = kernel function
- h = bandwidth
- n = total number of facilities
- $x_{i}$ = coordinates of facilities

This method effectively identifies the spatial clustering and areas of high institutional density which provide insights into accessibility and coverage. This spatial visualization serves as a foundation for analyzing imbalances and optimizing the distribution of institutions to enhance regional equity in the health and eldercare services in the region.

### 3.4 Comparative analysis and cross-validation of spatial methods

To ensure the reliability and robustness of the spatial analysis results, we conducted a cross-validation of the outcomes generated by the four methods used in this study: Ripley’s K-function, Geographic Concentration Index (G), Imbalance Index (S), and Kernel Density Estimation (KDE).

Overall, the findings were consistent across all methods, reinforcing the conclusion that the distribution of medical and older adult care institutions in Wuhan is spatially clustered and imbalanced.

- Ripley’s K-function revealed significant spatial clustering for all institution types, especially within central districts.
- Geographic Concentration Index (G) supported this finding by indicating values well above the uniform threshold (G > 27.73), confirming strong concentration.
- Imbalance Index (S) values greater than 0 for all categories quantified the spatial disparity, particularly among secondary hospitals.
- KDE analysis visually demonstrated these patterns, with high-density clusters observed in core urban areas and sparse coverage in peripheral districts.

These results converge to suggest that high-level medical institutions are disproportionately concentrated in central areas, while outer districts remain underserved. Importantly, no contradictory patterns were observed among the methods. The triangulation of statistical and visual approaches enhances the robustness and credibility of the spatial inequity assessment.

## 4 Results

### 4.1 Multidistance spatial cluster analysis (Ripley’s K function)

**Figure 3:**
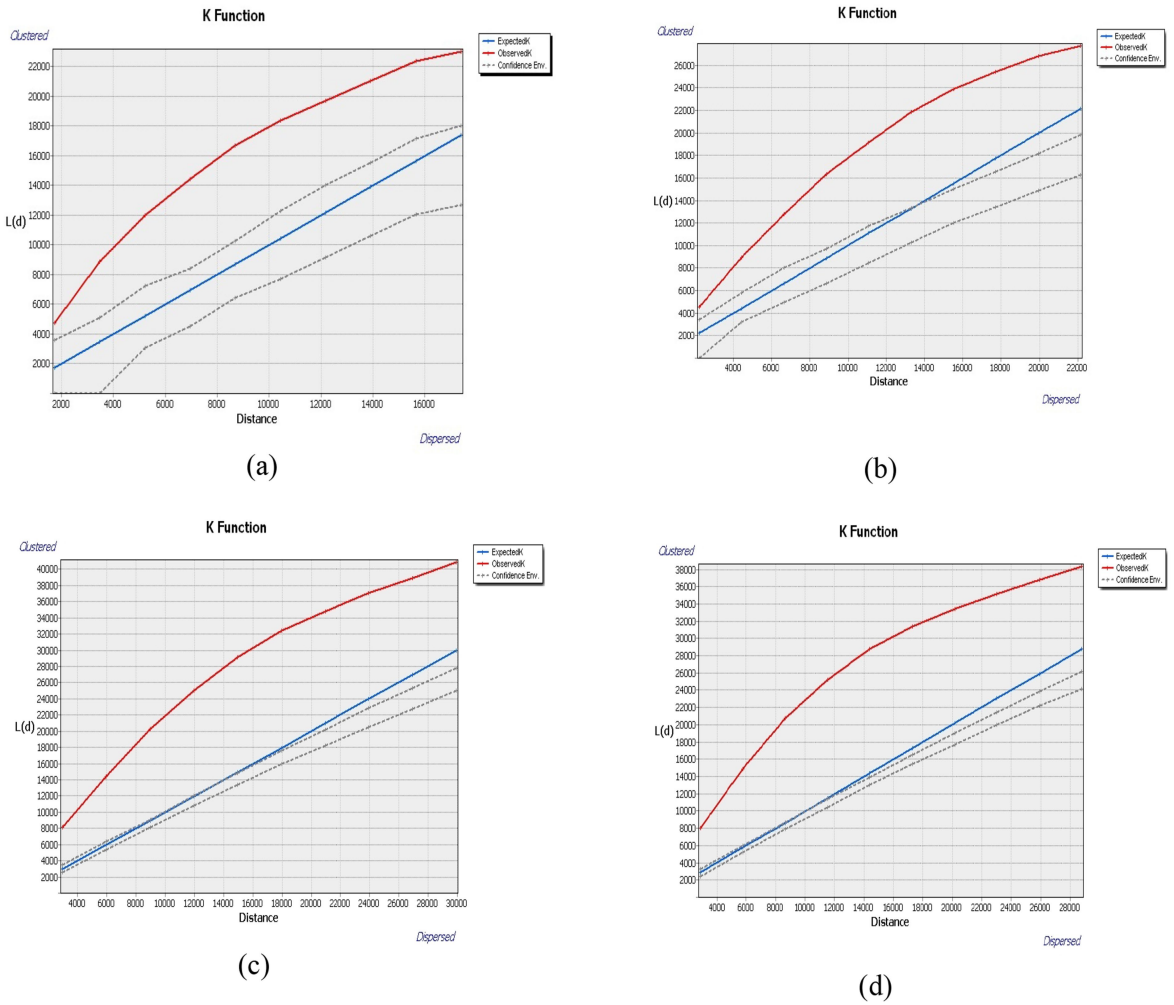
Results of Ripley’s K for each category of institutions. **(a)** Tertiary medical facility. **(b)** Secondary medical facility. **(c)** Community health center. **(d)** Senior care institution.

In this study, Ripley’s k function analysis was conducted with a 99% confidence interval, starting from an initial distance of 0 meter and increasing incrementally by 2,000-meters. The results reveal the spatial distribution patterns for the four categories of institutions: (a) tertiary hospitals, (b) secondary hospitals, (c) community health service centers, (d) senior care institutions as shown in Figure 3.

Across all categories, the observed *K*-values consistently exceeded both the expected *K* values and the HiConfEnv values across all four institution types as shown in Figures 3a–d confirming statistically significant spatial clustering. This indicates that all institution types are non-randomly and densely distributed within the urban core.

These findings indicates that tertiary hospitals, secondary hospitals, community health service centers, and older adult care institutions in Wuhan are not randomly distributed but exhibit a high degree of spatial concentration, aligning with established urban development patterns and demographic demands. Such clustering may have implications for accessibility and equitable distribution of health and eldercare services in the region. The initial distance (0 meters) and incremental step (2,000 m) for Ripley’s K-function analysis were chosen based on Wuhan’s urban structure and the typical service coverage of medical and older adult care facilities. These values align with urban density conditions and facility catchment areas used in similar spatial studies (44, 45). The 2,000-meter step allows the detection of both micro and macro-level clustering across different urban contexts. This range effectively captures spatial interactions within the average movement radius of older adult populations and urban healthcare seekers.

**Figure 4:**
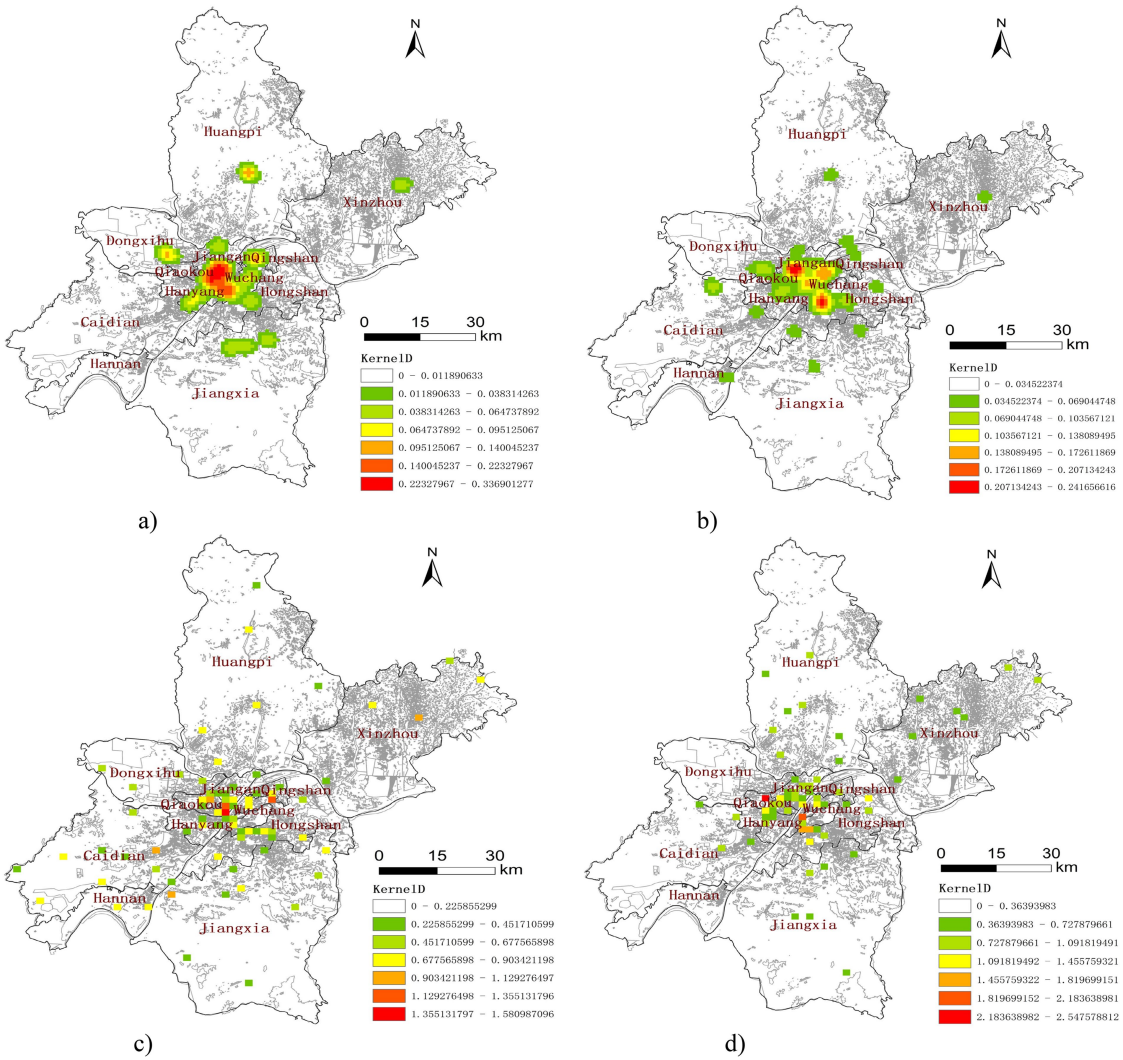
Kernel density maps of institutions in Wuhan. **(a)** Tertiary medical facility. **(b)** Secondary medical facility. **(c)** Community health center. **(d)** Senior care institution.

To complement spatial pattern analysis, we assessed the distribution of institutions relative to older adult population size across districts. Table 3 (detailed in Supplementary Table S3) shows the number of medical and older adult care institutions per 10,000 older adult individuals. Results indicate that some districts with a relatively low number of institutions (e.g., Jiangxia, Xinzhou) also have high older adult population density, reinforcing concerns about access inequity. Figure 4 visualizes these discrepancies, further illustrating the misalignment between supply and demand.

**Table 3:** Geographical concentration index and imbalance indices of various institutions in Wuhan.

Type of Institution	*G*	*S*
Tertiary medical institutions	31.77051	0.322581
Secondary medical institutions	34.1731	0.384921
Medical institutions above secondary	31.56635	0.321918
Community health service center	29.1175	0.19187
Senior care institutions	30.17925	0.262585

### 4.2 Geographic concentration index (G) and imbalance index (S)

The spatial clustering and imbalance in the distribution of healthcare and older adult care institutions are not random but rather the result of several systemic and policy-driven factors. First, historical urban development and land-use planning in Wuhan have favored the concentration of high-quality infrastructure-including tertiary hospitals and eldercare centers-within the central business districts along the Yangtze River. These areas benefit from higher population density, better transportation networks, and greater government investment, leading to resource agglomeration.

The results of the geographical concentration index (G) and imbalance index (S) are presented in Table 3. First, using, we obtain the *G* values and $G_{0}$ = 27.74. The result indicates that the *G* values for all the facilities in categories-tertiary hospitals (a), secondary hospitals (b), community health service centers (c), and senior care institutions (d) exceed $G_{0}$. This confirms a significant spatial concentration of institutions across Wuhan, particularly for high-level medical facilities.

**Figure 5:**
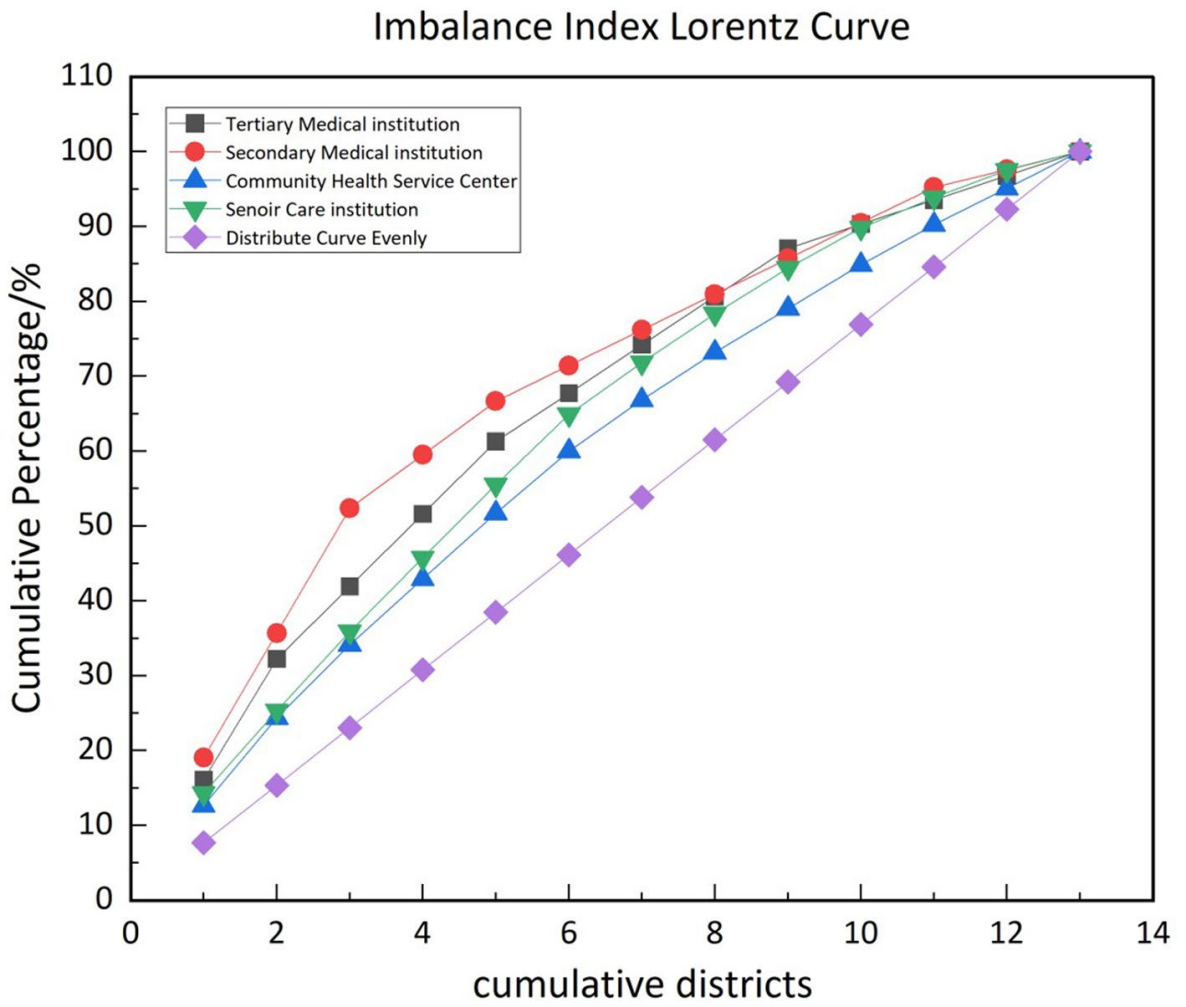
Lorenz curves of spatial imbalance for different types of institutions.

The Imbalance Index (S) values for all categories are also above zero, indicating a notable spatial imbalance. Secondary hospitals exhibit the highest imbalance (*S* = 0.385), while community health centers show the lowest (*S* = 0.192), suggesting relatively better spatial equity at the primary care level. This imbalance is visually represented in Figure 5, which shows that secondary hospital exhibits the highest spatial imbalance among the four studied categories. In contrast community health service centers demonstrate the most balanced spatial distribution.

These findings are consistent with prior research and underscore the need for strategies to mitigate geographic disparities in service availability, particularly for vulnerable populations relying on equitable access to health and eldercare services.

### 4.3 Kernel density analysis

Medical institutions of different levels and scales exhibit distinct service radii, which influence their accessibility and spatial distribution. In this study, kernel density analysis was employed using differentiated distance thresholds tailored to the various types of institutions analyzed. For tertiary hospitals, a distance threshold of 10,000 meters was employed. The secondary hospitals were assigned a threshold of 5,000 meters. The distance threshold for community health centers and senior care institutions were set at 2000 meters. This threshold determination considered two critical factors: the imperative for timely access to medical services particularly for older adult populations, and the service capacity of secondary hospitals.

Through the implementation of these differentiated thresholds, the analysis aims to achieve a more precise representation of the service coverage and accessibility patterns, ensuring a nuanced representation of the spatial distribution of healthcare and senior care resources. This nuanced approach enhances the kernel density analysis by considering the hierarchical nature of the healthcare system and the specific needs of older adult individuals, providing reliable framework for assessing accessibility.

The results of the kernel density analysis of the various institutions in Wuhan (Figure 4) reveals that the tertiary and secondary hospitals (Figures 4a,b) are highly concentrated in central districts such as Jiang’an district, Jianghan district, Qiaokou, Hanyang, and Wuchang core areas along the Yangtze River. Peripheral districts like Jiangxia, Huangpi, Caidian, and Xinzhou show limited coverage, suggesting lower accessibility.

Community health centers and senior care institutions (Figures 4c,d) demonstrate broader coverage but still exhibit core-periphery disparities. While their spatial reach is better than higher-level hospitals, underserved gaps remain in outlying districts.

This pattern underscores the uneven spatial allocation of healthcare and senior care resources, emphasizing the need for targeted interventions to address accessibility gaps in less served districts.

## 5 Discussion

This study employs a combination of spatial analytical methods to provide a comprehensive insight of the spatial distribution characteristics of medical and older adult care institutions in Wuhan. The results of Ripley’s K function analysis shows that the observed *K* values for all four categories of institutions consistently exceeded the expected *K* values and high confidence envelope values, indicating statistically significant spatial clustering. The geographic concentration index (G) supports this trend, with all *G* values surpassing the theoretical threshold $G_{0}$, demonstrating a pronounced tendency toward spatial concentration. Similarly, the imbalance index (S), with values exceeding 0 for all type of institutions, reflect an uneven distributing of resources. The observed clustering and imbalance in service distribution directly reflect the concerns raised by spatial justice theory, where unequal geographic access can lead to systemic exclusion. The concentration of high-level facilities in urban cores limits equity of access for suburban and rural older adult populations, demonstrating how physical location intersects with service availability to produce spatial inequity.

Second, the kernel density analysis complements these global indicators by visually illustrating the spatial disparities. The results identify an urban–rural imbalance, with most medical and senior care institutions concentrated in urban districts along the Yangtze River, Such as Jiang’an, Jianghan, and Wuchang, while peripheral and rural districts, including Hanyang, Jiangxia, and Xinzhou, experience resource scarcity. This skewed distribution reflects a clustering of high-quality medial resources in core urban areas and a relative neglect region, impacting accessibility and equity in healthcare service delivery.

Finally, this multimethod methodological framework enables us to gain a comprehensive understanding of the spatial distribution characteristics of medical and senior care resources, providing a scientific basis for subsequent policy-making and resource optimization.

Wuhan is geographically divided by the Yangtze River into eastern and western parts. The urban layout of Wuhan has developed along both sides of the Yangtze River, extending toward the periphery. Owing to factors such as the water system, urban framework, and traffic planning, Wuhan has evolved into a polycentric city rather than a central city in the economic sense. Even within the core urban districts, the majority of secondary and tertiary medical institutions creating challenges in accessibility and equitable distribution. Such clustering poses significant hurdles for the integration of healthcare and older adult care services, particularly in rural areas where resource is sparse. These institutions are predominantly concentrated in the Jiang’an, Jianghan, and Wuchang districts on both sides of the Yangtze River.

These findings reveal the spatial imbalance in the distribution of medical and senior care institutions in Wuhan, particularly the significant disparity in high-quality medical resources between urban and rural areas. This imbalance may lead to insufficient accessibility of medical resources for residents, especially older adult individuals, in rural and urban fringe areas. Addressing these disparities is critical for promoting equitable healthcare access, particularly in light of the “Healthy China 2030” initiative, which emphasizes universal access to public services across all life stages. Aligning with spatial justice principles, future planning should ensure that policy measures actively address geographic disparities in service provision-not only by increasing supply but by redistributing resources equitably based on need and accessibility gaps. These findings align with and directly support the goals outlined in “Healthy China 2030,” which emphasizes equitable access to basic public health services, especially for vulnerable groups such as the older adult. However, our spatial analysis reveals persistent mismatches between older adult population needs and the spatial distribution of service facilities-particularly in peri-urban and rural districts. To meet the objectives of “Healthy China 2030,” urban planning departments must integrate population data and spatial equity principles into their decision-making processes. This means not only increasing facility counts but redistributing resources based on need-adjusted metrics, such as facilities per older adult capita and travel-time accessibility.

While spatial analysis highlights geographic clustering and inequality, the distribution of healthcare and older adult care institutions in Wuhan is also shaped by several non-spatial structural factors.

- Economic inequality across districts influences infrastructure development. High-income urban districts such as Wuchang and Jiang’an benefit from stronger fiscal capacity, enabling greater public investment in healthcare and eldercare infrastructure. In contrast, outlying districts like Xinzhou and Huangpi lack comparable financial resources, which constrains both public and private service development.
- Variations in pension and health insurance coverage affect service demand and investment attractiveness. Districts with larger working-age or retired populations under stable pension schemes generate higher demand and better payment capacity, attracting private sector participation. Conversely, less economically active districts face demand-side constraints, disincentivizing investment.
- Return-on-investment concerns drive resource clustering in high-density zones. Private investors are more likely to establish facilities in urban centers with better transport, higher older adult population concentration, and greater revenue potential. This market-driven logic, while efficient for business, exacerbates spatial inequality in access.

These non-spatial influences intersect with geographic factors to produce the unequal patterns observed in this study. Thus, addressing equity in older adult service provision requires not only spatial planning but also fiscal rebalancing mechanisms and targeted subsidies to correct market failures in underserved areas.

As individuals age, the risk of disease increases due to the natural decline in bodily functions (51). Older people commonly experience various chronic conditions such as hypertension, diabetes, and respiratory diseases. The use of multiple medications is also prevalent among older adult individuals, which can lead to issues of medication misuse and abuse. Consequently, ensuring the adequacy of medical services becomes a crucial consideration for older adult when they are choosing a nursing home (52, 53). In reality, the quality processes implemented in healthcare facilities and by staff members directly affect quality-of-care outcomes. Maximizing the quality of service for older individuals involves establishing and maintaining quality processes of care (54). With the progress of urbanization, the concentration of population and resources has become more pronounced. Many rural workers have migrated to urban areas, depleting the rural workforce and creating urgent concerns regarding rural aging in China. High-quality medical resources tend to be concentrated in and around urban centers, so rural towns and remote areas have limited access to these resources. The uneven distribution of healthcare resources due to spatial agglomeration further restricts the access of rural aging populations to high-quality healthcare services.

In China, there are two main approaches to integrating healthcare and older adult care. The first is the embedded models, where older adult care facilities are established with in medical institutions, and contractual arrangements, wherein medical and older adult care institutions cooperate through service agreements. For administrative reasons and other reasons, the older adult care sector is managed by the Civil Affairs Ministry, whereas the medical care sector falls under the jurisdiction of the Ministry of Health. Contractual arrangements are more common as they allow for looser institutional associations and technology-driven synergistic relationships through cooperation agreements. From a supply perspective, the integrated model of medical and nursing care for older adult individuals in China is still facing challenges.

This study also enables a comparative assessment of the two primary models of healthcare–older adult care integration currently implemented across Wuhan: the embedded model and the contractual model. The embedded model, where older adult care services are co-located within healthcare institutions such as community health centers or hospitals, is more common in central districts with stronger health infrastructure. This model enhances care continuity and user satisfaction but requires higher investment and physical co-location.

In contrast, the contractual model involves independent medical and older adult care institutions working through referral agreements. It is more prevalent in peripheral and rural districts due to flexible implementation but often results in fragmented care and logistical barriers.

According to some scholars, this sector has been institutionally integrated but not yet functionally integrated (55). Compared with an embedded association, a contractual association is a looser institutional association, and this loose institutional association is less stable. The COVID-19 pandemic has further impacted business collaboration between older adult care and medical institutions, leading to a standstill in many cases due to political influences. In Wuhan, approximately one-tenth of the total number of senior care institutions (23 institutions) have adopted the embedded model of medical and older adult care integration. The majority of senior care institutions operate under contract-based models. Compared with large healthcare providers, geriatric care providers are mostly small-scale and have minimal economic impact. As a result, many do not have direct connections with large healthcare providers. Instead, they establish service agreements with nearby community healthcare centers. Moreover, a geriatric care provider often maintains relationships with several healthcare providers simultaneously, forming a complex network. Fragmented medical services and limited access to high-quality medical resources are common issues faced by individual older adult individuals during their aging process (56) and the uneven adsorption of health information system often influenced by hospital size and financial capacity, further complicates service integration (57). Technical information is often maintained independently between medical institutions and between medical institutions and senior care institutions (58), hindering the continuity and consistency of medical services received by older adult individuals (59). Applying validated assessment framework, similar to those employed in other healthcare areas like intimate partner violence screening (60), emphasizes the importance of the methodological rigor in evaluating and improving service delivery systems.

Improved access to older adult care institutions and resources can greatly benefit older individuals by reducing transportation costs, providing timely physical assistance, meeting their basic living needs (61), and ultimately contributing to longer life expectancies and improved well-being. However, the primary challenge in older adult care involves older individuals’ ability to financially afford it. Upon retirement, older adult have fewer opportunities for participation in economic activities, which results in reduced earning capacity and limited consumption ability. An analysis of China’s pension security system reveals significant inequalities in benefit distribution, both cross-sectionally and longitudinally (62, 63). Cross-sectionally, pension benefits vary widely among participants in different sectors and regions. Longitudinally, the growth rates of pension benefits are diverged over time, widening the gap between high and low recipients. These trends indicate increasing disparities in China’s pension system, potentially in terms of older adult welfare and social equity. In China, most older adult individuals bear the costs of pensions directly from their own pockets, and health insurance coverage does not fully cover the total cost of the medical services they require. The Chinese government primarily invests in older adult care by subsidizing the construction of infrastructure, providing beds, and supporting the operation of older adult care institutions. However, the subsidies provided directly to older adult themselves are minimal (55). Consequently, the vacancy rate of older adult care beds continues to increase annually despite the growth in the number of available beds. This economic inequality-driven demand for public older adult care and public health services significantly affects equity in accessing these services.

## 6 Limitations

While this study provides important insights into spatial equity and service distribution, several limitations of the GIS-based spatial analysis must be acknowledged.

First, the use of projected coordinate systems (e.g., Gauss-Krüger or Web Mercator) may introduce spatial distortion, especially when analyzing large-scale distances or working at the city fringe. Although Wuhan’s urban area limits this risk, minor projection-induced errors may affect accuracy in distance-based clustering analyses such as Ripley’s K-function.

Second, geocoding limitations may introduce location-based inaccuracies. Although most institution addresses were validated using Baidu Maps and cross-referenced with official registries, some records required manual correction or approximation, particularly for rural institutions. This may affect the precision of spatial point pattern analysis.

Third, parameter selection in spatial models (e.g., KDE bandwidths and K-function distance thresholds) can affect output sensitivity. We mitigated this by conducting sensitivity analyses and aligning parameter values with prior research and known service radii.

Lastly, while we aimed for data completeness, the exclusion of unverifiable or unregistered institutions may have introduced selection bias. However, triangulation across multiple methods (K, G, S, KDE) and population-adjusted ratios supports the robustness of our findings despite these limitations.

## 7 Conclusion

Population aging poses long-term economic implications, impacting global labor supply, capital accumulation, and gross national product. Addressing these challenges requires the modernization of aging governance systems which are essential for strengthening national framework and enhance institutional capacities (64). The process of modernization varies significantly across different regions, cities, and communities within China resulting in diverse patterns of social governance within localized contexts (65). Despite these variations, the government models must adhere to principles of adaptability and responsiveness.

In the public health sector, the policymakers should prioritize the equitable provision of medical and senior care services in a manner that is accessible, non-discriminatory and barrier-free. This study reveals that the spatial distribution of medical and senior care institutions in Wuhan reflects significant clustering and imbalance. High-quality medical resources are concentrated in certain areas, while many rural and peri-urban areas face limited accessibility to healthcare and older adult care facilities. This disparity impacts older adult individuals ability to have equitable access to high-quality medical services (66). Therefore, addressing the imbalances and inequalities in service provision should be the starting point for necessary policy adjustments.

To improve accessibility, it is critical to provide medical services close to older adult care facilities where the aging population is concentrated is essential. This approach enhances accessibility and reduces travel time for older adult residents, especially those in suburban and rural areas.

Based on the findings of spatial clustering, urban–rural disparities, and population-adjusted service gaps, the following policy recommendations are proposed:

- Redistribute Resources Using Need-Based Allocation Ratios: Urban planning departments and health authorities should adopt data-driven service allocation models that prioritize districts with high older adult populations and low facility-per-capita ratios. These ratios should inform funding and facility development priorities at both city and district levels.
- Expand Embedded Integration Models in Underserved Areas: Encourage the establishment of embedded older adult care units within existing community health centers, especially in peripheral districts like Xinzhou and Caidian. These models reduce travel burdens and support aging-in-place, aligning with the Healthy China 2030 goals.
- Implement Financial and Regulatory Incentives for Rural Facility Investment: Local governments should introduce subsidies, tax relief, and fast-track land use approvals for private and public investments in older adult care and healthcare facilities in rural areas. This will help correct the profit-driven bias toward urban cores.
- Mandate Spatial Equity Impact Assessments in Future Urban Development Plans: Urban expansion and facility planning should include mandatory spatial equity evaluations using GIS-based models to ensure equitable access in all new developments.
- Enhance Cross-Departmental Coordination: Strengthen collaboration between the Health Commission and Civil Affairs Bureau to improve integrated planning and avoid fragmented or duplicated service allocation.

To better capture spatial heterogeneity within Wuhan, a multi-scale spatial analysis was conducted by categorizing districts into three levels: urban core (e.g., Jiang’an, Wuchang, Qiaokou), suburban (e.g., Caidian, Dongxihu), and peripheral/rural areas (e.g., Xinzhou, Huangpi). As shown in Figure 4, institutional densities are highest in the urban core, with significantly lower service coverage in suburban and rural districts. This pattern is further confirmed in Table 2, which reports the number of healthcare and older adult care facilities per 10,000 older adult residents across the three zones as detailed in Supplementary Tables S4, S5.

In conclusion, prioritizing equitable access to medical and senior care services for the aging population must remain a central focus of healthcare policies. By promoting integration between medical resources and senior care facilities and addressing the disparities in resource distribution are essential to optimizing the overall healthcare system’s efficiency. By taking these steps, policymakers can significantly improve the quality of life for the older adult population in Wuhan and set an example for the other regions facing similar challenges in China.

## Data Availability

The datasets used and analyzed during the current study are not publicly available due to confidentiality agreements with the Wuhan Civil Affairs Bureau and the Wuhan Health Administration Committee. However, anonymized data may be made available from the corresponding author upon reasonable request and with permission from the respective authorities.

## Supplementary material

Supplementary Table S1 Geocoded location dataset of healthcare and elderly care institutions in Wuhan.

Supplementary Table S2 Calculated values of the Imbalance Index and Geographic Concentration Index for healthcare and elderly care institutions in Wuhan.

Supplementary Table S3 Geospatial dataset of medical and elderly care institutions in Wuhan.

Supplementary Table S4 Location and address dataset of elderly care institutions in Wuhan.

Supplementary Table S5 District-wise distribution of healthcare and elderly care institutions in Wuhan, including counts of tertiary hospitals, secondary hospitals, community health centers, and senior care facilities.

## References

[1] Saif-Ur-Rahman K Saif-Ur-Rahman KM Mamun R Eriksson E He Y Hirakawa Y. Discrimination against the elderly in health-care services: a systematic review. Psychogeriatrics. (2021) 21:418–29. doi: 10.1111/psyg.12670, PMID: 33634922

[2] Wilson T Temple J Brijnath B Utomo A McDonald P. The ageing of Asian migrant populations in Australia: projections and implications for aged care services. Asian Popul Stud. (2022) 18:61–86. doi: 10.1080/17441730.2021.1953689

[3] Huang Y Meyer P Jin L. Neighborhood socioeconomic characteristics, healthcare spatial access, and emergency department visits for ambulatory care sensitive conditions for elderly. Prev Med Rep. (2018) 12:101–5. doi: 10.1016/j.pmedr.2018.08.015, PMID: 30233997 PMC6138954

[4] Xu A Zare H Dai X Xiang Y Gaskin DJ. Defining hospital community benefit activities using Delphi technique: a comparison between China and the United States. PLoS One. (2019) 14:e0225243. doi: 10.1371/journal.pone.0225243, PMID: 31747421 PMC6867695

[5] Zhenzhen Z. The population decline in China: a global perspective. Popul Res. (2023) 47:3.

[6] Fanghua G Sanhui T. Internet intervention system for elderly hypertensive patients based on hospital community family edge network and personal medical resources optimization. J Med Syst. (2020) 44:95–12. doi: 10.1007/s10916-020-01554-1, PMID: 32193696

[7] Andrews G Duff C. Understanding the vital emergence and expression of aging: how matter comes to matter in gerontology's posthumanist turn. J Aging Stud. (2019) 49:46–55. doi: 10.1016/j.jaging.2019.04.002, PMID: 31229218

[8] Aronson L. Healthy aging across the stages of old age. Clin Geriatr Med. (2020) 36:549–58. doi: 10.1016/j.cger.2020.06.001, PMID: 33010893

[9] China, N.B.o.S.o. (2023). The seventh national population census bulletin (no. 5) – age composition of the population, in Popul stud. National Bureau of Statistics of China. Available online at: https://www.stats.gov.cn/english/PressRelease/202105/t20210510_1817190.html

[10] Yanfeng G Liejun W Wenmeng F. Challenges and strategic choices for healthy aging in China. Manag World. (2020) 36:86–96.

[11] Ding Y Cai X Ou Y Liang D Guan Q Zhong W. The burden of diabetes in the southeastern coastal region of China from 1990 to 2019 and projections for 2030: a systematic analysis of the 2019 global burden of disease study. Diabetes Metab Res Rev. (2025) 41:e70031. doi: 10.1002/dmrr.70031, PMID: 39831738

[12] Yip W Fu H Chen AT Zhai T Jian W Xu R. 10 years of health-care reform in China: progress and gaps in universal health coverage. Lancet. (2019) 394:1192–204. doi: 10.1016/S0140-6736(19)32136-1, PMID: 31571602

[13] Xiaofan M Haifeng Z Ziyi G Ao S. Study on spatial differentiation of population aging and mismatch of endowment resources in Xining City. World Regional Studies. (2021) 30:213. doi: 10.3969/j.issn.1004-9479.2021.01.2019516

[14] Huang X Gong P White M. Study on spatial distribution equilibrium of elderly care facilities in downtown Shanghai. Int J Environ Res Public Health. (2022) 19:7929. doi: 10.3390/ijerph19137929, PMID: 35805586 PMC9266222

[15] Lilleheie I Debesay J Bye A Bergland A. Informal caregivers’ views on the quality of healthcare services provided to older patients aged 80 or more in the hospital and 30 days after discharge. BMC Geriatr. (2020) 20:1–13. doi: 10.1186/s12877-020-1488-1, PMID: 32164569 PMC7068939

[16] Pearce J Witten K Bartie P. Neighbourhoods and health: a GIS approach to measuring community resource accessibility. J Epidemiol Community Health. (2006) 60:389–95. doi: 10.1136/jech.2005.043281, PMID: 16614327 PMC2563982

[17] He AJ Tang VF. Integration of health services for the elderly in Asia: a scoping review of Hong Kong, Singapore, Malaysia, Indonesia. Health Policy. (2021) 125:351–62. doi: 10.1016/j.healthpol.2020.12.020, PMID: 33422336

[18] Cheng Y Gao S Li S Zhang Y Rosenberg M. Understanding the spatial disparities and vulnerability of population aging in China. Asia Pac Policy Stud. (2019) 6:73–89. doi: 10.1002/app5.267

[19] ZhouYLiYZhuXMaLDr. Medical and old-age care integration model and implementation of the integrated care of older people (ICOPE) in China: opportunities and challenges. J Nutr Health Aging. (2021) 25:720–3. doi: 10.1007/s12603-021-1595-5, PMID: 34179923

[20] Guagliardo MF. Spatial accessibility of primary care: concepts, methods and challenges. Int J Health Geogr. (2004) 3:1–13. doi: 10.1186/1476-072x-3-3 14987337 PMC394340

[21] Wang F Luo W. Assessing spatial and non-spatial factors for healthcare access: towards an integrated approach to defining health professional shortage areas. Health Place. (2005) 11:131–46. doi: 10.1016/j.healthplace.2004.02.003, PMID: 15629681

[22] Pan J Deng Y Yang Y Zhang Y. Location-allocation modelling for rational health planning: applying a two-step optimization approach to evaluate the spatial accessibility improvement of newly added tertiary hospitals in a metropolitan city of China. Soc Sci Med. (2023) 338:116296. doi: 10.1016/j.socscimed.2023.116296, PMID: 37879131

[23] Lee SWH Mak VSL Tang YW. Pharmacist services in nursing homes: a systematic review and meta-analysis. Br J Clin Pharmacol. (2019) 85:2668–88. doi: 10.1111/bcp.14101, PMID: 31465121 PMC6955407

[24] Cinnamon J Schuurman N Crooks VA. A method to determine spatial access to specialized palliative care services using GIS. BMC Health Serv Res. (2008) 8:1–11. doi: 10.1186/1472-6963-8-140 18590568 PMC2459163

[25] Haixian LZZFX. Spatial matching evaluation of supply and demand of elderly service facilities based on multi-source data—Nanjing City as an example. Mod Urban Res. (2022). doi: 10.3390/su142316183

[26] Zhu L Zhong S Tu W Zheng J He S Bao J. Assessing spatial accessibility to medical resources at the community level in Shenzhen, China. Int J Environ Res Public Health. (2019) 16:242. doi: 10.3390/ijerph16020242, PMID: 30654500 PMC6352203

[27] Busingye D Pedigo A Odoi A. Temporal changes in geographic disparities in access to emergency heart attack and stroke care: are we any better today? Spat Spatiotemporal Epidemiol. (2011) 2:247–63. doi: 10.1016/j.sste.2011.07.010, PMID: 22748224

[28] Blanchard IE Doig CJ Hagel BE Anton AR Zygun DA Kortbeek JB. Emergency medical services response time and mortality in an urban setting. Prehosp Emerg Care. (2012) 16:142–51. doi: 10.3109/10903127.2011.614046, PMID: 22026820

[29] Eisenberg MS Bergner L Hallstrom A. Cardiac resuscitation in the community: importance of rapid provision and implications for program planning. JAMA. (1979) 241:1905–7. doi: 10.1001/jama.1979.03290440027022 430772

[30] Chen L Chen T Lan T Chen C Pan J. The contributions of population distribution, healthcare resourcing, and transportation infrastructure to spatial accessibility of health care. Inquiry. (2023) 60:00469580221146041. doi: 10.1177/00469580221146041, PMID: 36629371 PMC9837279

[31] McLafferty SL. GIS and health care. Annu Rev Public Health. (2003) 24:25–42. doi: 10.1146/annurev.publhealth.24.012902.141012, PMID: 12668754

[32] Johnson O Diggle P Giorgi E. Dealing with spatial misalignment to model the relationship between deprivation and life expectancy: a model-based geostatistical approach. Int J Health Geogr. (2020) 19:1–13. doi: 10.1186/s12942-020-00200-w 32131836 PMC7057663

[33] Di X. Urban-rural difference of the impact of the accessibility of community elderly care services on the quality of life of the elderly from the perspective of social support. Research Square (Research Square) (2024). doi: 10.21203/rs.3.rs-3987210/v1

[34] Tsai C-F Hwang WS Lee JJ Wang WF Huang LC Huang LK. Predictors of caregiver burden in aged caregivers of demented older patients. BMC Geriatr. (2021) 21:59–9. doi: 10.1186/s12877-021-02007-1, PMID: 33446114 PMC7809883

[35] Wang Y Gonzales E Morrow-Howell N. Applying WHO’S age-friendly communities framework to a national survey in China. J Gerontol Soc Work. (2017) 60:215–31. doi: 10.1080/01634372.2017.1292980, PMID: 28409710

[36] Sixsmith J Sixsmith A Fänge AM Naumann D Kucsera C Tomsone S. Healthy ageing and home: the perspectives of very old people in five European countries. Soc Sci Med. (2014) 106:1–9. doi: 10.1016/j.socscimed.2014.01.006, PMID: 24524960

[37] Pavlovic S Jovanović R. Geographical index of concentration as an indicator of the spatial distribution of tourist attractions in Belgrade. Turizam. (2021) 25:7553. doi: 10.5937/turizam25-27553

[38] Van Egeraat C Haifeng Z Ziyi G Ao S. A measure for identifying substantial geographic concentrations. Pap Reg Sci. (2018) 97:281–301. doi: 10.1111/pirs.12241

[39] Kim SW Haghparast-Bidgoli H Skordis-Worrall J Batura N Petrou S. A method for measuring spatial effects on socioeconomic inequalities using the concentration index. Int J Equity Health. (2020) 19:9–13. doi: 10.1186/s12939-019-1080-5, PMID: 31937314 PMC6958664

[40] Yang D-H Goerge R Mullner R. Comparing GIS-based methods of measuring spatial accessibility to health services. J Med Syst. (2006) 30:23–32. doi: 10.1007/s10916-006-7400-5, PMID: 16548411

[41] Seaman DE Powell RA. An evaluation of the accuracy of kernel density estimators for home range analysis. Ecology. (1996) 77:2075–85. doi: 10.2307/2265701

[42] Węglarczyk S. Kernel density estimation and its application ITM web of conferences (2018). 23 p.

[43] Gao F Kihal W le Meur N Souris M Deguen S. Does the edge effect impact on the measure of spatial accessibility to healthcare providers? Int J Health Geogr. (2017) 16:46–16. doi: 10.1186/s12942-017-0119-3, PMID: 29228961 PMC5725922

[44] Ripley BD. Modelling spatial patterns. J R Stat Soc Series B Stat Methodol. (1977) 39:172–92. doi: 10.1111/j.2517-6161.1977.tb01615.x

[45] Yates LA Brook BW Buettel JC. Spatial pattern analysis of line-segment data in ecology. Ecology. (2022) 103:e03597. doi: 10.1002/ecy.3597, PMID: 34816432

[46] Florence P.S. Investment, location, and size of plant: A realistic inquiry into the structure of British and American industries (1948).

[47] Crafts N Klein A. Spatial concentration of manufacturing industries in the United States: re-examination of long-run trends. Eur Rev Econ Hist. (2021) 25:223–46. doi: 10.1093/ereh/heaa027

[48] Harris R Moffat J Evenhuis E Martin R Pike A Sunley P. Does spatial proximity raise firm productivity? Evidence from British manufacturing. Camb J Reg Econ Soc. (2019) 12:467–87. doi: 10.1093/cjres/rsz017

[49] Zhao H Yue L Jia Z Su L. Spatial inequalities and influencing factors of self-rated health and perceived environmental hazards in a metropolis: a case study of Zhengzhou City, China. Int J Environ Res Public Health. (2022) 19:7551. doi: 10.3390/ijerph19127551, PMID: 35742800 PMC9224377

[50] Hart T Zandbergen P. Kernel density estimation and hotspot mapping: examining the influence of interpolation method, grid cell size, and bandwidth on crime forecasting. Policing. (2014) 37:305–23. doi: 10.1108/pijpsm-04-2013-0039

[51] Wang Z Wang S Lin H Wang C Gao D. Prevalence of hypertension and related risk factors in older Chinese population: a meta-analysis. Front Public Health. (2024) 12:1320295. doi: 10.3389/fpubh.2024.1320295, PMID: 38686031 PMC11056525

[52] Marino M de Belvis AG Tanzariello M Dotti E Bucci S Colotto M. Effectiveness and cost-effectiveness of integrated care models for elderly, complex patients: a narrative review. Don’t we need a value-based approach? Int J Care Coord. (2018) 21:120–39. doi: 10.1177/2053434518817019

[53] van Rijckevorsel-Scheele J Willems R Roelofs P Koppelaar E Gobbens R Goumans M. Effects of health care interventions on quality of life among frail elderly: a systematized review. Clin Interv Aging. (2019) 14:643–58. doi: 10.2147/CIA.S190425, PMID: 31040654 PMC6453553

[54] Cleland J Hutchinson C Khadka J Milte R Ratcliffe J. What defines quality of care for older people in aged care? A comprehensive literature review. Geriatr Gerontol Int. (2021) 21:765–78. doi: 10.1111/ggi.14231, PMID: 34258840

[55] Feng Z Glinskaya E Chen H Gong S Qiu Y Xu J. Long-term care system for older adults in China: policy landscape, challenges, and future prospects. Lancet. (2020) 396:1362–72. doi: 10.1016/S0140-6736(20)32136-X, PMID: 34338215

[56] Zhang YM Dongmei. Research on the evaluation of the operation effect of combined medical and health care services in the context of healthy China—a survey based on Yinchuan City. Scient Res Aging. (2018) 6:47–61.

[57] Luo J Ahmad SF Alyaemeni A Ou Y Irshad M Alyafi-Alzahri R. Role of perceived ease of use, usefulness, and financial strength on the adoption of health information systems: the moderating role of hospital size. Humanit Soc Sci Commun. (2024) 11:516. doi: 10.1057/s41599-024-02976-9

[58] Du CZ Luheng. From ‘fragmented operation’ to ‘holistic governance’: a study on the innovation of the path of smart elderly service supply. Study Pract. (2020):92–101.

[59] Li Q You T Chen J Zhang Y Du C. LI-EMRSQL: linking information enhanced Text2SQL parsing on complex electronic medical records. IEEE Trans Reliab. (2023) 73:1280–90. doi: 10.1109/TR.2023.3336330

[60] Li Y Wang G Chen J Xia Q Chen K Ou S. Evaluation of the measurement properties of intimate partner violence screening instruments for the general population: a COSMIN-based international systematic review. PLoS One. (2024) 19:e0310297. doi: 10.1371/journal.pone.0310297, PMID: 39541374 PMC11563433

[61] Tao Z Cheng Y. Modelling the spatial accessibility of the elderly to healthcare services in Beijing, China. Environ Plan. (2019) 46:1132–47. doi: 10.1177/2399808318755145, PMID: 40503249

[62] Kornreich Y Vertinsky I Potter PB. Consultation and deliberation in China: the making of China's health-care reform. China J. (2012) 68:176–203. doi: 10.1086/666583

[63] Guo AG Danan. The impact of health care accessibility on health of the elderly from the perspective of health inequality—an empirical analysis based on CLHLS data. Populat Dev. (2020) 26:60–9.

[64] Lin B. New Progress of China's elderly service policy since the 18th party congress Journal of the Party School of the Central Committee of the Communist Party of China (National School of Administration) (2021). 25 p.

[65] Wu XHZCP He R. Technologies of technology governance: practices, types and their adaptation logics – a multi-case study based on community governance in Nanjing. J Public Adm. (2022) 19:107–20.

[66] Yin W Liu S. Study on spatial distribution and accessibility of the elderly care institutions in Shanghai. Mod Urban Res. (2021) 6:17–23.

